# 3D Propolis-Sodium Alginate Scaffolds: Influence on Structural Parameters, Release Mechanisms, Cell Cytotoxicity and Antibacterial Activity

**DOI:** 10.3390/molecules25215082

**Published:** 2020-11-02

**Authors:** Kubra Aranci, Muhammet Uzun, Sena Su, Sumeyye Cesur, Songul Ulag, Al Amin, Mehmet Mucahit Guncu, Burak Aksu, Sevgi Kolayli, Cem Bulent Ustundag, Jorge Carvalho Silva, Denisa Ficai, Anton Ficai, Oguzhan Gunduz

**Affiliations:** 1 Center for Nanotechnology & Biomaterials Application and Research (NBUAM), Marmara University, 34722 Istanbul, Turkey; aranci36@gmail.com (K.A.); senasu01@gmail.com (S.S.); sumeyye-cesur@hotmail.com (S.C.); ulagitu1773@gmail.com (S.U.); 2 Department of Bioengineering, Graduate School of Natural and Applied Sciences, Yıldız Technical University, 34220 Istanbul, Turkey; 3 Department of Textile Engineering, Faculty of Technology, Marmara University, 34722 Istanbul, Turkey; islamalamin6710@gmail.com; 4 Department of Metallurgical and Materials Engineering, Faculty of Technology, Marmara University, 34722 Istanbul, Turkey; 5 Institute of Health Sciences, Department of Microbiology, Marmara University, Maltepe, 34854 Istanbul, Turkey; mmguncu@gmail.com; 6 Department of Medical Microbiology, School of Medicine, Marmara University, Maltepe, 34854 Istanbul, Turkey; drbaksu@gmail.com; 7 Department of Chemistry, Faculty of Sciences, Karadeniz Technical University, 61080 Trabzon, Turkey; skolayli61@yahoo.com; 8 Department of Bioengineering, Faculty of Chemical and Metallurgical Engineering, Yıldız Technical University, 34220 Istanbul, Turkey; cbustundag@gmail.com; 9 Departamento de Física and CENIMAT/i3N, Faculdade de Ciências e Tecnologia, Universidade Nova de Lisboa, 2829-516 Caparica, Portugal; jcs@fct.unl.pt; 10 Department of Inorganic Chemistry, Physical Chemistry and Electrochemistry, Faculty of Applied Chemistry and Materials Science, University POLITEHNICA of Bucharest, Gh Polizu Street 1-7, 011061 Bucharest, Romania; denisa.ficai@upb.ro; 11 National Centre for Micro- and Nanomaterials, University POLITEHNICA of Bucharest, Independentei St. 313, 060042 Bucharest, Romania; 12 Department of Science and Engineering of Oxide Materials and Nanomaterials, Faculty of Applied Chemistry and Materials Science, University POLITEHNICA of Bucharest, Gh Polizu Street 1-7, 060042 Bucharest, Romania; 13 Academy of Romanian Scientists, Ilfov st. 3, 050094 Bucharest, Romania

**Keywords:** 3D printing, propolis, sodium alginate, tissue scaffold, wound treatment

## Abstract

In this study, the main aim was to fabricate propolis (Ps)-containing wound dressing patches using 3D printing technology. Different combinations and structures of propolis (Ps)-incorporated sodium alginate (SA) scaffolds were developed. The morphological studies showed that the porosity of developed scaffolds was optimized when 20% (*v*/*v*) of Ps was added to the solution. The pore sizes decreased by increasing Ps concentration up to a certain level due to its adhesive properties. The mechanical, swelling-degradation (weight loss) behaviors, and Ps release kinetics were highlighted for the scaffold stability. An antimicrobial assay was employed to test and screen antimicrobial behavior of Ps against *Escherichia coli* and *Staphylococcus aureus* strains. The results show that the Ps-added scaffolds have an excellent antibacterial activity because of Ps compounds. An in vitro cytotoxicity test was also applied on the scaffold by using the extract method on the human dermal fibroblasts (HFFF2) cell line. The 3D-printed SA–Ps scaffolds are very useful structures for wound dressing applications.

## 1. Introduction

Additive manufacturing (AM) or three-dimensional (3D) printing is an advanced technology that is used for obtaining as a matter the output of a three-dimensional model [1,2]. This technology allows the manufacturing of customizable, biocompatible, patient-specific, quite comprehensive, mechanically stable, and rapidly fabricated products in medical fields [2,3]. Notably, 3D-printed tissue scaffolds are popular in regenerative medicine and tissue engineering applications in medicine [4]. Normally, tissue scaffold manufacturing methods are divided into two classes: traditional production methods and additive manufacturing methods [5]. The difference between additive manufacturing techniques and traditional production methods is that the former provide the opportunity to produce materials in a very short time without the need for extra equipment, no matter how complex the structure is [6]. In traditional production methods, the pores, pore sizes and architecture of tissue scaffolds cannot be easily controlled. This situation results in inconsistent and non-ideal 3D scaffolds. To overcome such problems, researchers have suggested the use of 3D printing methods to produce customized scaffolds with controlled pore size, pore structure and scaffold architecture [7]. Therefore, an innovative 3D method was preferred in this study to control the scaffold parameters. There are many studies related to 3D-printed tissue scaffolds that are based on synthetic and natural polymers in the literature [3,4,8,9]. However, SA (sodium alginate)-based scaffolds are popular in tissue applications.

SA is the most preferred natural anionic polymer that is used for biomedical applications due to its biocompatibility, low toxicity and low cost [10]. The application of SA as scaffolds can be in the form of hydrogels, films/membranes and nanofibers [11]. For instance, Straccia et al. prepared alginate hydrogels coated with chitosan to delay the release of hydrophilic molecules from the alginate matrix [12]. Kamoun et al. showed that poly (vinyl alcohol)–alginate hydrogels loaded with sodium ampicillin as a topical antibiotic could be excellent membranes for wound care [13]. Furthermore, alginate can be printed three-dimensionally to manufacture scaffolds. Luo et al. prepared 3D-printed bone forming peptide-1 (BFP-1)-loaded alginate scaffolds for enhanced bone regeneration [14]. Liakos et al. studied antimicrobial and antifungal essential oils (EOs) dispersed in SA films to change the antimicrobial activity of alginate polymer. In scaffolds, protection of wounds against unwanted bacteria is a vital situation. Therefore, a useful substance addition could be an excellent option to prevent the colonization of the wound by bacteria [15].

Propolis (Ps) or bee glue is a resinous substance collected by honey bees from leavess, exudates, buds, etc. of different plants to build, isolate and protect beehives [16]. Generally, it is collected from pine, willow, oak, elm, nectar and pollen materials [17]. The Ps sources, season, collecting time, different locations (geographical differences), illumination, altitude and producing methods can play an essential role in changing the physical and chemical properties of Ps [18]. There are more than 300 compounds in Ps, according to the literature [19]. Antimicrobial and antioxidant effects of Ps come from phenolic compounds. The antimicrobial activity of Ps has been especially widely investigated [20]. Ps has antibacterial [21,22,23], antiviral [24], antifungal [25], antioxidant [26], anti-inflammatory [27], antitumor [28], anesthetic [20] and analgesic properties. Therefore, it is a good candidate for use as a therapeutic substance in the biomedical field and for wound treatment [29]. The wound healing process is composed of four stages: hemostasis, inflammation, proliferation (granulation-contraction) and remodeling steps. Ps plays a role in the initial phase of the wound repair such as hemostasis and inflammation. It is responsible for stimulating the expression of transforming growth factor-β (TGF-β) that regulates nearly all cellular events [30].

In the literature, there are few studies related to Ps and SA. Keskin et al. produced alcohol-free Ps–SA microcapsules by the encapsulation method [16]. They displayed the controlled release of Ps active components. In another study, Candido et al. obtained neomycin, an antibiotic that is very popular in diabetic ulcers, and Ps-incorporated SA hydrogel as a treatment dressing for diabetic ulcers [31]. They tried both of the hydrogel and the microparticle form of the SA. The microparticles were produced using the dripping extrusion method, and the hydrogels were prepared through the casting method. In both alginate-based wound dressing forms, Ps and neomycin were used. The researchers showed that all samples are good candidates to use as an absorbent in wound treatments. Furthermore, Juliano et al. studied polymeric films containing Ps [32]. They prepared the Ps and added agar, alginate and alginate-chitosan based films. They also performed antimicrobial tests against *Escherichia coli*, *Staphylococcus aureus* and *Candida albicans* strains. They suggested that the alginate and alginate-chitosan films were proposed as a vehicle for the buccal delivery of Ps extracts at the end of the study. As seen in the literature on 3D printing techniques, SA and Ps are well known due to their several benefits. The 3D printing method is employed to develop Ps-based SA structures for wound dressing applications.

This study aims to construct biocompatible, 3D-printed SA scaffolds by adding Ps extract to improve biological and wound healing properties. With the help of the high antimicrobial activity of propolis, we aimed to minimize unwanted infection situations which are frequently encountered during the wound healing process. The optimized Ps-incorporated SA solutions were printed with different combinations and parameters. The rheological properties of the obtained printing inks such as viscosity, density and surface tension were investigated. Furthermore, the mechanical and the morphological features of the 3D-printed scaffolds also were characterized. The antibacterial assay of 3D-printed, biofunctional SA–Ps scaffolds were investigated. The cytocompatibility of produced SA scaffolds with different amounts of Ps was studied using the MTT test. As mentioned, there are limited studies related to SA–Ps for wound treatment.

## 2. Materials and Methods

### 2.1. Chemicals

Sodium alginate (SA) with an average molecular weight of 216.121 g/mol was purchased from Sigma-Aldrich (Istanbul, Turkey). Calcium chloride dihydrate (CaCl_2_.2H_2_O) was purchased from Merck (Darmstadt, Germany) for use as crosslinker. The Ps extract was kindly provided from SBS (Scientific Bio Solutions) Incorporated Company, Istanbul, Turkey). PBS (phosphate-buffered saline) pH 7.4 and pH 2.0 solutions were bought from ChemBio Laboratory Research (Istanbul, Turkey).

Mueller Hinton agar (CM0337B, Oxoid, Thermo Fisher, Basingstoke, UK) and Mueller Hinton broth media (CM0405B, Oxoid), and 2 μg and 10 μg ampicillin containing disks (CT0002B and CT0003B, Oxoid) were obtained for antibacterial activity assays. *Escherichia coli* ATCC^®^ 25922™ and *Staphylococcus aureus* ATCC^®^ 29213™ standard strains were used for antibacterial testing.

The evaluation of potential cytotoxicity of SA–Ps scaffolds was performed according to the ISO 10,993 standard (Biological evaluation of medical devices) “Part 5—Tests for in vitro cytotoxicity”, using the extract method and the HFFF2 cell line (human dermal fibroblasts). Cells were cultured in DMEM (Dulbecco′s Modified Eagle′s Medium with 1 g/L glucose, with stable glutamine, with sodium pyruvate, Biowest #L0066) supplemented with penicillin (100 U/mL), streptomycin (100 µg/mL) (Invitrogen, #15140122) and 10% FBS (Fetal Bovine Serum, S. America origin, Biowest, #S1810). Cells were seeded on a 96 well plate at a density of 20k cells/cm^2^ the day before placing the extracts in contact with cells and were incubated at 37 °C in a 5% CO_2_ humidified atmosphere incubator (Sanyo MCO-19AIC (UV)).

### 2.2. Preparation of Solutions

The SA and SA–Ps solutions in different concentrations were prepared. The summary of each solution’s content is given in Table 1. Firstly, the SA polymer solution was dissolved in distilled water and 20 mL of 4.5% (*w*/*v*) SA solution was prepared at room temperature.

The SA solution was stirred at 1000 rpm for nearly 4 h to obtain a clear appearance. Secondly, different concentrations of SA–Ps solution were prepared. The different amounts were taken from the prepared 4.5% (*w*/*v*) SA solution and liquid Ps extract in various amounts were added dropwise to SA solution. The SA–Ps solutions were prepared as 10%, 20% and 40% (*v*/*v*) concentrations of Ps. They were named as: Sample 1 that is pure SA, Sample 2 (10% *v/v* Ps content), Sample 3 (20% *v/v* Ps content) and Sample 4 (40% *v/v* Ps content) respectively. The SA concentration stayed constant as 4.5% (*w*/*v*). All solutions’ total volume was 5 mL. This new combination of SA and Ps-incorporated solutions were mixed in a stirrer for 30 min. Then, 1% (*w*/*v*) of CaCl_2_.2H_2_O was used as a crosslinker.

### 2.3. Design and 3D Printing of Scaffolds

**Figure 1:**
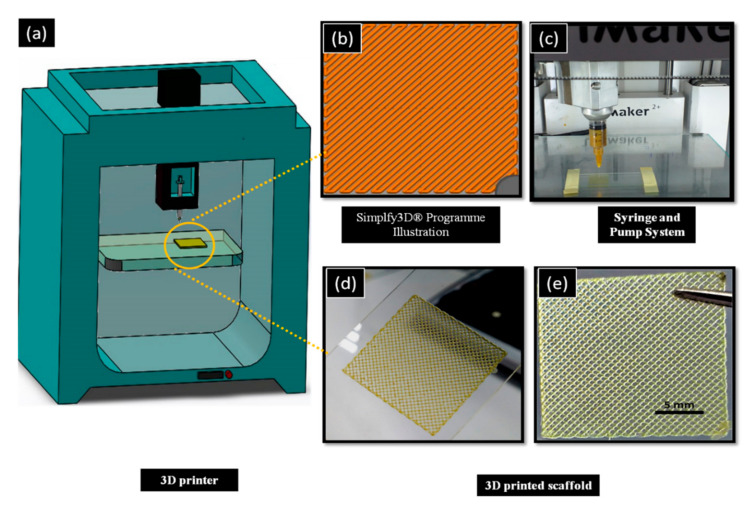
Schematic drawing of the experiment. (**a**) 3D printer. (**b**) Syringe pump system of machine. (**c**) Simplyf3D program illustration of scaffolds. (**d**) 3D-printed scaffold in glass slide before crosslinking. (**e**) After crosslinking of 3D-printed scaffolds.

A schematic drawing of the 3D printing process is shown in Figure 1. The scaffolds were designed as square 20 × 20 × 1 mm^3^ dimensions with an average porosity ~75% using CAD Software (SolidWorks^®^.) The roughly drawn scaffold structure was converted into an STL (stereolithography) file that is the required file format for 3D printing and was transferred to Simplify3D^®^ software. The designed scaffolds were printed using a modified extrusion 3D printer (Ultimaker^2+^, Netherlands) which manipulated from the computer-aided pump part. The digital syringe pump was used to feed the solutions. The nozzle diameter was 0.35 mm. The printing speed was 650 mm/min for control SA scaffolds, however 550 mm/min speed was chosen for Ps-added SA scaffolds. The scaffold layer was 20 for all scaffolds. The infill pattern was rectilinear with 45-degree infill direction. According to prior studies, square pore morphology has a higher compressive strength and higher modulus than other pore morphologies [33]. Therefore, the pattern was chosen as rectilinear and square pore morphology. These parameters were determined as a result of optimization studies.

The prepared Ps-based SA solution was transferred to a 10 mL syringe for 3D printing. Firstly, the coordinates of the syringe were adjusted, and secondly, the glass slide was placed on the table of the 3D printer. After giving the necessary parameters to the program and centering the 3-axis of the printer, SA-based scaffolds were printed. The CaCl_2_ crosslinker solution was used to consolidate the printed scaffold and for easier removal. The scaffolds were exposed to the CaCl_2_ for 15 min. After completely drying, the printed material was removed from the glass slide carefully by the help of a lancet blade. During the experiment, the temperature was set at room temperature, but a fan was used to accelerate proper drying.

### 2.4. Characterizations

#### 2.4.1. Physical Properties Characterization

The density, viscosity and surface tension of the solutions were determined. The densities of control SA and Ps-incorporated SA solutions were determined by using a 10 mL standard pycnometer. The surface tension of the solutions was measured with a force tensiometer (Sigma 703D, Attension, Darmstadt, Germany). The viscosity of solutions was determined by a digital viscometer (DV-E, Brookfield, Middleboro, MA, USA). Each equipment was calibrated before the tests and all samples were tested three times.

#### 2.4.2. Fourier Transform-Infrared Spectroscopy (FT-IR)

Chemical characterization of scaffolds was performed using a Fourier Transform Infrared (FT-IR) Spectrometer (Jasco, FT-IR 4700). The FT-IR spectra of samples were founded at the scanning range of 400 and 4000 cm^−1^ and a resolution of 4 cm^−1^.

#### 2.4.3. Scanning Electron Microscopy (SEM)

The morphological characterization of scaffolds was also performed with scanning electron microscopy (SEM, EVA MA 10, ZEISS, Pleasanton, CA, USA). The samples’ surfaces were coated with gold before imaging to obtain a conductive surface. The average pore size and distribution of the pores for each sample were determined with the help of imaging software (Olympus AnalySIS, Waltham, MA, USA).

#### 2.4.4. Mechanical Properties of the Scaffolds

The tensile test of the scaffolds was carried out by a tensile test machine (SHIMADZU, EZ-LX, Beijing, China). The thickness and length of the scaffolds were measured by a digital micrometre (Mitutoyo). The three samples from SA and SA–Ps scaffolds were measured. All samples were placed uniaxial to the appropriate section of the device from their upper and lower positions.

#### 2.4.5. In Vitro Release Profile of Ps

**Figure 2:**
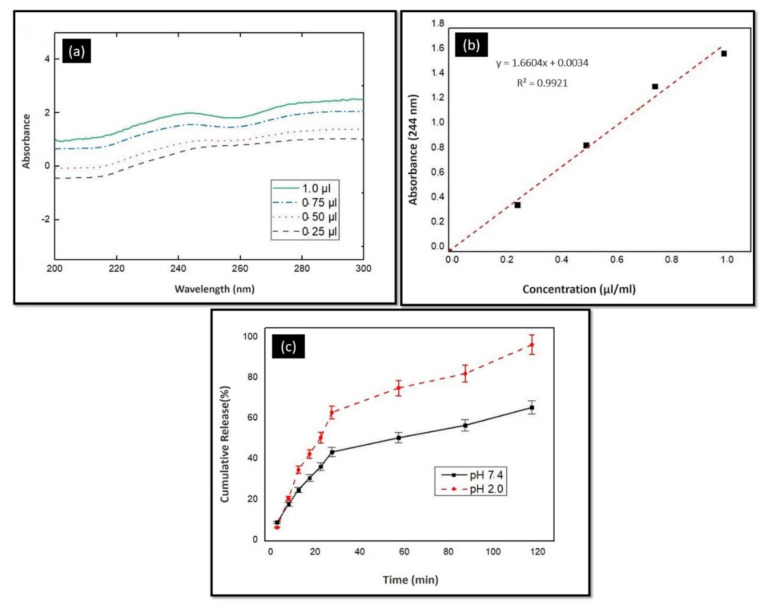
(**a**) Absorbance values according to different dilution ratios for the standard calibration curve. (**b**) Standard calibration curve of Ps solution. (**c**) Release profile of 3D-printed Ps-incorporated SA scaffolds in different pH mediums.

A standard calibration curve is a necessary graph for determining unknown concentrations. Therefore, this curve was prepared before starting the release test. The solutions containing different Ps ratios were prepared and measured spectrophotometrically by UV-1280 at 200–400 nm. The highest absorbance values and wavelength of Ps were determined to draw a standard calibration curve. The absorbance graphs of the solutions are shown in Figure 2a. The standard calibration curve was also drawn, as shown in Figure 2b.

The release profile of Ps from 3D-printed scaffold was determined in PBS (pH 2.0 and pH 7.4) buffer solutions. Sample 3 was chosen for the release test. The sliced scaffolds (9 mm × 9 mm) were placed into 2 mL of PBS buffer solution medium. Samples were incubated at 37 °C in a thermal shaker (BIOSAN TS-100) at 250 rpm. The release profile of Ps was performed in time intervals of 5 min until the first 30 min and then up to 120 min at half hour intervals. Then, 1 mL of solution was taken from the PBS solution and replaced with 1 mL of fresh PBS solution. The saturated PBS solutions were measured spectrophotometrically by UV-1280 (SHIMADZU, 200–400 nm). The cumulative releasing percentage was calculated by the help of the standard calibration curve of Ps, as shown in Figure 2c.

#### 2.4.6. Ps Release Kinetics

In order to investigate the Ps release mechanism from 3D-printed scaffolds, Ps release profiles were fitted to two popular kinetic models, Higuchi and Korsmeyer—Peppas (Power Law) equations. (Equations (1) and (2)).

Higuchi equation defines a linear dependence of the active fraction released per unit of the square of time.

Q = K_h_t (1)

where Q is the fractional amount of Ps released at time t and K_h_ is release rate constant. 

The equation of Korsmeyer–Peppas is

Q = Kt^n^ (2)

where Q is the fractional amount of Ps released at time t, K is the kinetic constant and n is the diffusion exponent which is indicative of the drug release mechanism.

The interpretation of the release mechanism by the n value depends on the geometry of the matrix. For a cylindrical system, when n approximates to 0.45, Fickian diffusion is implied and 0.45 < n < 1 refers to the non-Fickian diffusion.

#### 2.4.7. Swelling and Degradation Behaviors of Scaffolds

The scaffolds were sliced equally and placed into 2 mL PBS solution (pH = 7.4). They were incubated in a thermal shaker at 37 °C. The swollen scaffolds’ weights were measured after removing excess water by filter paper. The swelling test was measured at half-minute intervals for 300 min. Furthermore, the swelling ratio was calculated using Equation (3) [34]:

(3) $$\mathrm{SR}=\frac{W_{t}-W_{0}\;}{W_{0}}$$

where *W_t_* is swollen samples and *W*_0_ is the samples’ weight before swelling.

The degradation (weight loss) of scaffolds was performed in a plastic eppendorf filled with 2 mL PBS solution (pH = 7.4). The degradation tests were also conducted in a thermal shaker at 37 °C. The swollen scaffolds were removed from PBS medium, washed with distilled water and dried at room temperature for 2.5 h at predetermined time intervals. The degradation was determined by measuring weight loss (%) by using Equation (4):

(4) $$\;\mathrm{Weight}\;\mathrm{loss}\;\left(\%\right)=\frac{W_{1}-W_{2}}{W_{1}}\;\times \;100$$

where W_1_ and W_2_ are the weights of the scaffolds before and after degradation, respectively.

#### 2.4.8. Antibacterial Assay

Overnight cultures of *E. coli* ATCC^®^ 25922™ and *S. aureus* ATCC^®^ 29213™ in Mueller-Hinton broth were used to prepare bacterial suspensions within the same broth and adjusted to 0.5 McFarland turbidity standard (1–2 × 10^8^ CFU/mL). Resulting bacterial suspensions were inoculated on Mueller-Hinton agar plates by using an automated plate inoculator. Disks (5 mm in diameter) were cut from Ps-containing scaffolds and held for 1 h under UV light (254 nm) for sterilization. Sterilized disks were placed on the surface of bacteria-inoculated Mueller-Hinton agar plates with sterile forceps. Ampicillin-containing disks (2 and 10 μg) were used as a positive control for *S. aureus* ATCC^®^ 29213™ and *E. coli* ATCC^®^ 25922™, respectively. Plates were incubated at 37 °C for 18 h and growth inhibition zones around disks were measured and evaluated.

#### 2.4.9. Cell Cytotoxicity

The extracts were prepared using a mass of material to the volume of the culture medium of 20 mg/mL for the SA scaffolds and 5 mg/mL for the SA with Ps scaffolds. The latter scaffolds have a mass ratio of Ps to SA of 0.3:1.0. Scaffolds were sterilized by exposure to UV radiation from the UV lamp of a biological safety cabinet (ESCO Labculture II) for 30 min each side. The extraction conditions were 48 h at 37 °C. Then, the cell culture medium was aspirated and the extracts placed in contact with cells. In this study, HFFF2 cell lines (human fetal foreskin fibroblast) were used. Extracts were serially diluted by a factor of two in order to prepare equivalent concentrations of 10, 5 and 2.5 mg/mL for the SA scaffold extracts and 2.5, 1.25, 0.63, 0.31, 0.16, 0.08 and 0.04 mg/mL. For each concentration, 4 replicates were prepared. Negative (viable cells) and positive (cells in a cytotoxic environment) controls were established by culturing cells with normal medium and medium supplemented with 10% dimethyl sulfoxide (DMSO), respectively. The extracts remained in contact with cells for 48 h. Finally, the cell population was evaluated using the resazurin assay [35]. All media were replaced by a 1:1 mixture of culture medium and a resazurin solution (0.04 mg/mL in PBS). After 2 h of incubation in the CO_2_ incubator, medium absorbance was measured at 570 nm and 600 nm with a microplate reader (Biotex ELX 800UV). 

#### 2.4.10. Statistical Analysis

All data were presented as mean ± standard deviation (SD). The statistical analysis was performed using SPSS software with a *p*-value ≤ 0.05 considered statistically significant. All measurements were performed in duplicates and their mean values were taken as the final result.

## 3. Results and Discussion

### 3.1. Synthesis Results

The optimum parameters of the solution for producing the 3D structure were investigated and the optimized solution was chosen according to its density, viscosity and surface tension properties. Notably, the viscosity of the solution is the most important parameter for the 3D printing process. A solution with high viscosity would possibly clog the needle tip and prevent the next layer printing. In contrast to high viscosity, if the solution viscosity is too low, the printed strands tend to spread. Thus the two adjacent layers join together and the next layers collapse due to merge. Therefore, the viscosity of the solutions must be adjusted appropriately and selected for 3D printing [36]. In addition to viscosity, surface tension is also an important parameter. There is a relationship between surface tension and droplet. If the surface tension of the resin or solution is too high, the solution will not be jetted through the nozzle. In contrast to this, if the surface tension is too low, the droplet shape would be changed and non-uniform droplets will exist instead of cohesive droplets [37].

The prepared SA and Ps-incorporated SA solutions’ viscosity, surface tension and density were measured. The effect of viscosity, density and surface tension on the scaffolds is demonstrated in Table 2.

All the 3D-printed scaffold images were in the same magnification. Sample 3 has the highest viscosity and surface tension compared to the other solutions according to the results. When the Ps content increases, the viscosity and surface tension increase simultaneously, as shown in Table 2.

As stated above, a certain level of increased viscosity provides a positive effect on printability [38]. Furthermore, the Ps is a resinous substance; therefore, a higher amount of Ps would increase the viscosity and surface tension of the solutions due to stickiness. Sample 4 was not appropriately produced owing to the high viscosity of the solution. The high viscosity solution causes printability problems [39]. For instance, the solution comes out of the machine infrequently, which causes destroyed pore shapes. Furthermore, filaments are thicker and close the pores due to the viscosity. The desired scaffold shape will not be obtained, as shown in Table 2. In addition to surface tension and viscosity parameters, there was no significant difference between each sample for density. The viscosity and surface tension of the optimized solution was chosen as Sample 3 (4.5% *w/v* SA-20% *v/v* Ps solution) for 3D printing of scaffolds. The production and experiments were carried out with these optimized parameters.

### 3.2. Characterization Results

#### 3.2.1. Fourier-Transform Infrared Spectroscopy (FT-IR)

**Figure 3:**
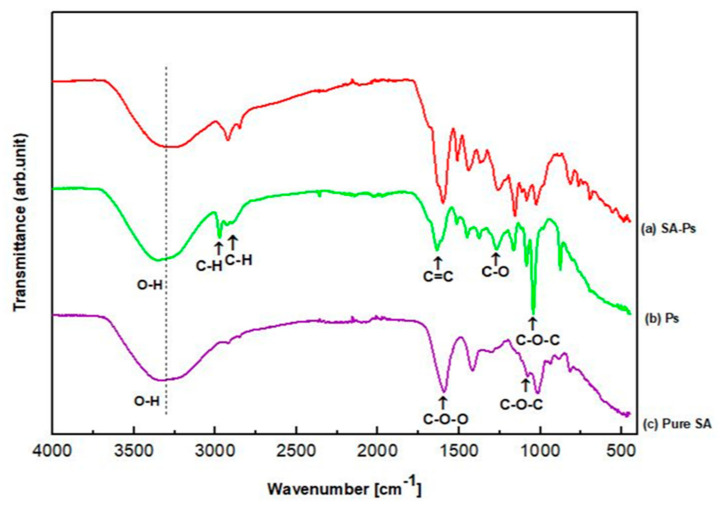
FT-IR spectrums of (**a**) SA with Ps tissue scaffolds, (**b**) only Ps solution and (**c**) pure SA solution.

FT-IR is a chemical analysis that is used to determine polymer (SA) and effective substance (Ps) interaction. Both FT-IR spectra of SA and SA–Ps scaffolds are shown in Figure 3. For pure SA, absorption bands are related to some functional groups such as hydroxyl, ether and carboxylic functional groups [40]. SA shows characteristic stretching vibrations of O-H peak at 3334 cm^−1^ and the literature also proves that this characteristic band is found in the range of 3000–3600 cm^−1^ [40,41]. Asymmetric stretching vibration of -COO- groups peak at 1593 cm^−1^, symmetric stretching vibration of -COO-groups peak at 1419 cm^−1^ and -C-O-C- stretching vibration peaks at 1078 cm^−1^ were obtained [42,43,44].

The FT-IR spectrum of Ps is shown in Figure 3b and it is nearly similar to pure SA, except in regards to the intensity of the peaks. For instance, Ps showed an O-H stretching vibration peak at 3344 cm^−1^ due to ethanol. The C-H stretching vibration peak at 2974 and 2927 cm^−1^ due to CH_2_ and CH_3_ bands and the C=C stretching vibration peak found at 1637 cm^−1^ due to aromatic ring deformations are also typical peaks of resins. However, according to the literature, these peaks would be probably related to stretching vibration of C=C and C=O groups with the flavonoids and amino acids. The C-H bending vibration and aromatic stretching vibration peaks were observed at 1450 cm^−1^. The C-H bending peak in CH_3_ was also seen at 1377 cm^−1^, but sometimes this peak was non-identified according to some studies [45]. The band at 1271 cm^−1^ would be probably associated with C-O group of polyols and also related to C-H bending. This peak is typical for resins. The C-O-C stretching and C-F stretching vibration peaks were obtained at 1146 cm^−1^. The C-O stretching peak from esters and C-O-C stretching vibration peak were found at 1086 cm^−1^. The peak at 1086 cm^−1^ can be related to secondary alcohol while the peak at 1043 cm^−1^ would be related to both primary and secondary alcohol. Finally, the peak at 878 cm^−1^ was frequently non-identified, but probably there is a relation of the C-C stretching vibration [45,46,47]. As shown in Figure 3, SA and Ps characteristic peaks were displayed for 3D-printed scaffolds. The integration of Ps and SA was successfully achieved according to these peak results.

#### 3.2.2. Scanning Electron Microscopy (SEM)

The size and structure of pores are essential factors for tissue scaffold applications. The three-dimensional porous scaffold provides cell adhesion and proliferation. Cells move easily to repair zones during the wound healing process thanks to these pores. Open and interconnected pores are necessary for mechanical support, cell nutrition, the permeability of gases and removal of toxic substances (by products) from tissues [48]. If the porosity is high, it provides more nutrient transport to tissues but results in lower structural density due to reduced mechanical stability [49]. Typically, it is challenging to choose optimal pore size values in tissue scaffolds due to different types of tissues and cell diversity. However, some optimum pore size ranges are proposed for different kinds of cells and tissues [50]. As a general rule, the size of the pores should be a few times larger than the cells which have to be accommodated inside these pores or to be penetrated to assure deep integration and new tissue formation. If the size of the pores is smaller than the size of the cells, the cells will not be able to populate the deeper part of the implant and only a surface integration can be assured. If the size of the pores is much larger, the mechanical properties decrease drastically and this is again a major shortcoming of these materials. For instance, Yannas et al. displayed that 20–125 µm pore size is optimal for skin regeneration [51]. The pore size of 100–400 µm is acceptable for bone tissues [52,53]. Salem et al. showed that a 40–150 µm pore size range is better for fibroblast binding [54]. Furthermore, porosity depends on the used material; therefore, a certain pore size range is challenging.

**Figure 4:**
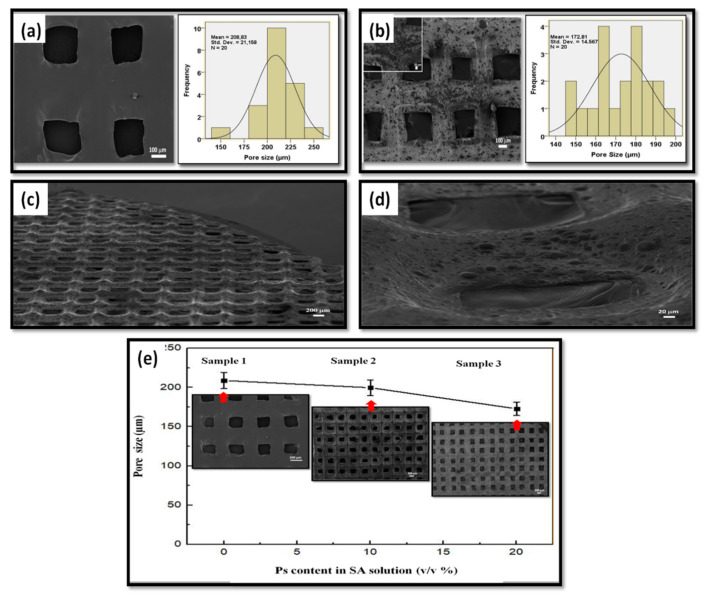
SEM images of 3D-printed SA and Ps-incorporated SA scaffolds. (**a**) SA scaffolds and pore size histogram (Sample 1). (**b**) Ps included SA scaffolds and pore size histogram (Sample 3) (**c**) SEM image of Sample 3 scaffold from different angle. (**d**) Magnification of the scaffold pores. (**e**) Pore size distribution according to propolis content.

The scaffolds’ SEM images, pore size histograms and pore size distribution according to propolis content are shown in Figure 4a–e. Results demonstrated that average pore size distributions of Sample 1 were 208 µm and the surface was smooth (Figure 4a). The average pore sizes for Sample 2 and Sample 3 were 202 µm and 172 µm, respectively, and surfaces were rough due to some agglomerated Ps powders, as shown in Figure 4e. The roughness is beneficial for cell attachment and proliferation [55,56]. When the Ps content increased, the pore sizes decreased. The decrease in average pore sizes can be explained due to the increasing volume of the dry content [57]. The adjacent filaments will be closer to each other. Therefore, the pores were smaller than Sample 1 (pure SA). Sutjarittangtham et al. showed that fiber diameters were decreased by the addition of different concentration of Ps. The fiber diameters were decreased from 140–190 nm to 60–70 nm due to higher Ps content [58]. It is also important to mention that when analyzing the surfaces of the strands there are no cracks or defects (such as, especially, micropores in the structure of strands or shrinking of the strands) which could affect the structural integrity, especially when exposed to tensile loading. In Figure 4c, a special topology of the 3D-printed scaffold can be seen, with the joints visualized as a hill while the middle of the strands are visualized as a valley. This hill-valley structuration along with the macroporous design are important because they will assure a better attachment of the cells and surrounding tissues, being well embedded into the surrounding tissue, until total resorption, because alginate usually can be resorbed in the body [59,60].

Typically, the scaffolds were designed as 20 × 20 × 1 mm^3^ (X, Y, Z dimensions). However, the dimensions were measured as 18 × 18 × 0.08 mm^3^ which means that, overall, a slight structural shrinking appears. The difference between the designed and produced scaffold dimensions may result from the crosslinking process with CaCl_2_ as well as due to a slight collapsing of the strands, but this is necessary to glue the strands from consecutive layers. Gao et al. studied the dual crosslinking effect on alginate hydrogel microfibers [61]. In this experiment, high CaCl_2_ concentration means high Ca cations and these ions interchange the alginate fibers in the “egg-box” structure due to sodium and calcium ions interaction. The alginate strands formed a cooperative binding structure which ensures the increase in alginate gel tightness. Therefore, the crosslinking can be shown as a reason for the shrinking of the dimensions by increasing the structure tightness [62].

#### 3.2.3. Tensile Test

Structural properties of porous scaffolds, such as pore sizes and porosity, are essential for their mechanical properties as well as for nutrient transport and they may affect their potential performance after introduction into the defect site [63]. A tensile test was performed to analyze the effects of mechanical properties on printed scaffolds. The tensile properties of all scaffolds were examined at room temperature, as shown in Table 3, with values of tensile strength and elongation at break.

The tensile strength results of the distribution for each of the three samples, as follows, respectively, were 3.92 ± 1.08 MPa, 2.42 ± 0.62 MPa and 2.33 ± 1.76 MPa. Strain at break percentages were 14.74 ± 1.22% for Sample 1, 20.57 ± 1.96% for Sample 2 and 25.00 ± 6.68% for Sample 3. These results are crucial in determining the flexibility of the product to achieve successful wound treatment [64]. The results clearly showed that the tensile strength of Sample 1 has a maximum tensile strength of 3.92 ± 1.08 MPa and minimum tensile value of 14.74 ± 1.22%, compared to the others. With the addition of different proportions of Ps to SA, tensile strength decreased from 3.92 MPa to 2.33 MPa. Elongation at break (%) increased from 14.74 to 25. The reason for this situation is that Ps has plasticizing properties [65]. When the effect of pore size on mechanical properties was examined, scaffolds having a pore size of 208 µm (Sample 1), 202 µm (Sample 2) and 172 µm (Sample 3) caused significant differences in the tensile strength values of the produced scaffolds. As Ps was incorporated into SA, it would normally be expected that the smaller the pore, the tighter the structure and the higher the tensile strength. This is because the bond structure of the pure SA polymer is stronger than the bond structure between SA–Ps, as the Ps particles do not act as a reinforcing agent but rather as a defect [66]. The good mechanical properties can be correlated with the SEM images and they can conclude that the lack of cracks and pores inside the strands as well as the lack of strand narrowing can assure good mechanical properties.

#### 3.2.4. In Vitro Release Tests

The release test of Ps was evaluated in 2 different media, one of them near an acidic value (pH = 2.0) and the other near a basic value (pH = 7.4) The samples were taken every five minutes for the first thirty minutes. After that, a sample was taken every thirty minutes until 120 min (2 h). As shown in Figure 2, there was a faster release in pH 7.4 medium than pH 2.0 buffer solution. The Ps was released nearly 96.76% at pH 2.0 within 2 h. However, the cumulative release of Ps reached up to almost 98.95% at pH 7.4 within 4 h. Normally, the release of Ps at pH 7.4 is 65.81% within 2 h. It seems that the release of Ps at pH 7.4 is a good candidate for controlled drug delivery systems. These results concur with the literature. Zhang et al. showed that 90% of Ps was quickly diffused into simulated gastric fluid (with pH close to 2.0) within 1 h and the rest of the Ps was diffused into simulated intestinal fluid (with pH close to nearly 7.0) due to hydrophilic phenolic compounds [67]. Juliano et al. showed that polyphenols slowly release and reach 96.3% value for maximum releasing after 2 h from SA capsules at pH 7.0 [31]. In another study, the fast release profile of Ps was observed at the beginning of the experiment. However, the in vitro release still was controlled despite Ps concentration increasing [18]. In our results, rapid and controlled release was also observed.

Wound healing is a complex process. There is a relationship between pH and wound milieu. All biochemical processes are affected by the pH of the media. Studies show that there is a shift toward an acidic pH forming during wound healing stages [68]. The continued release of drugs or effective substances during this pH change is a considerable state. Therefore, 3D-printed, SA–Ps tissue scaffolds show a rapid release profile even in acidic environments, which once again indicates their suitability for use as a wound dressing in wound treatments.

#### 3.2.5. Ps Release Kinetics

In vitro Ps release from 3D scaffolds was investigated in two different pH values and the release data were fitted to two different models to determine Ps release mechanisms. The correlation coefficients R^2^ obtained for each model are shown in Table 4. The highest R^2^ values (0.9822 and 0.9515) were obtained from Higuchi kinetics, which means that the release of Ps is controlled by diffusion through the polymeric scaffolds, obeying Fick’s laws (1st and 2nd). When Ps release fit to Korsmeyer–Peppas equation, R^2^ values were 0.9715 and 0.8633 in neutral and acidic media, respectively. It can be seen that the release mechanism of Ps follows Fickian diffusion by n ~ 0.45 only in a neutral medium. The Korsmeyer–Peppas kinetic is not suitable to use for the acidic medium because of the low R^2^. This may be due to the faster release of Ps from the SA matrix. Similar results were obtained in previous studies [69,70].

#### 3.2.6. Swelling and Degradation Tests

**Figure 5:**
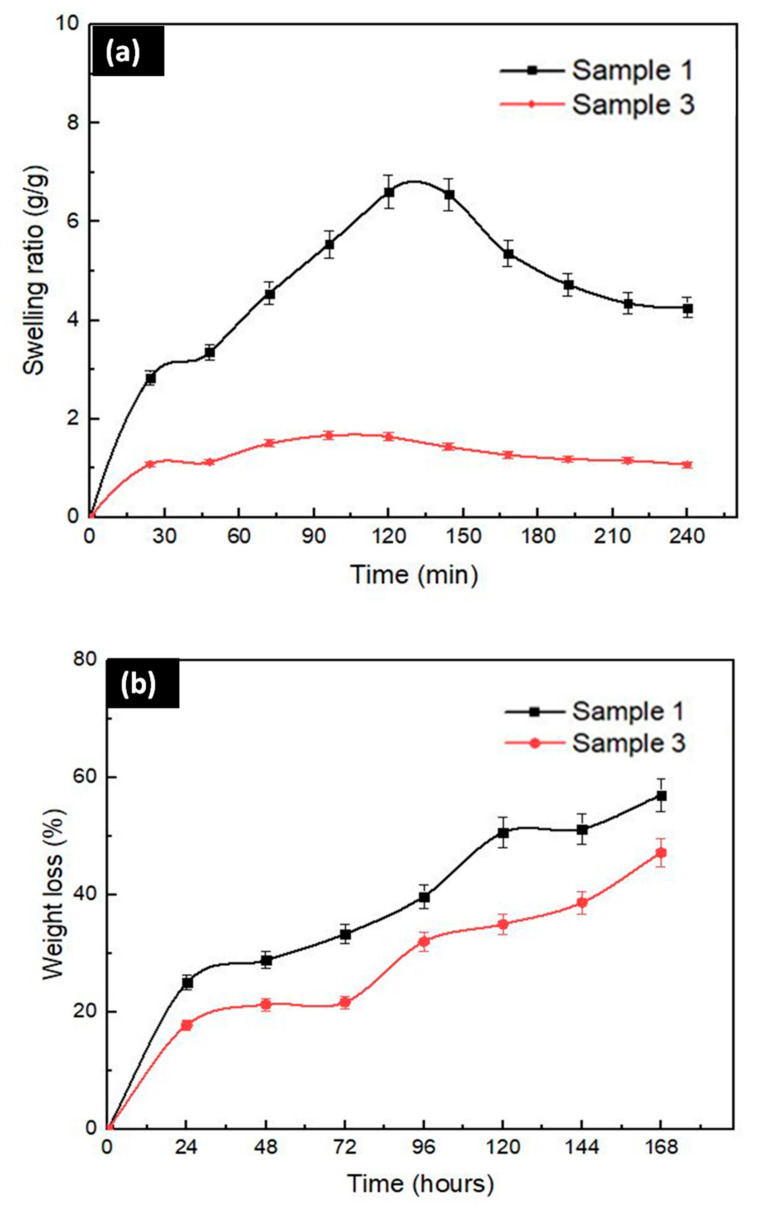
(**a**) Swelling and (**b**) degradation kinetics of 3D-printed Sample 1 and Sample 3 scaffolds in PBS 7.4 at 37 °C.

The water absorption or swelling ability of tissue scaffolds is an important parameter that allows bodily fluids, wound exudates, absorption and transfer of cell nutrients inside the scaffold. The swelling under physiological conditions should be controlled to prevent rapid degradation and mechanical weakening of tissue scaffolds [71]. As shown in Figure 5a, there is a high swelling ratio in the first phases of both samples (Sample 1 and Sample 3). This swelling is probably caused by the entrance of PBS to the scaffold fibers [57].

The swelling ratio of Sample 1 was more significant than the Sample 3 scaffold. Normally, it is known that alginate dressings have a high water uptake capacity [72,73]. However, there was a decrease in swelling with the addition of Ps into the SA scaffolds in this study. Oliveira et al. observed that Ps causes less space for water uptake by filling the pores, thereby reducing the stretching in the fibers with less water [57]. Candido et al. also showed that the addition of Ps and the interaction of SA chains with Ps result in low water absorption [74]. The swelling ratio is also correlated with the lower hydrophilic properties of Ps compared to alginate and in this case, practically, only SA adsorbed water leading to swelling and mass gain, at least for a while. After this, alginate started to dissolve and when the amount of dissolved SA is high enough, even the Ps particles are detached from the scaffolds and this can lead to more abrupt mass loss, for instance. Not only the addition of Ps but also the pore structure is one of the most important parameters affecting water uptake. A high porosity structure provides high water uptake [75,76]. The high swelling ratio in Sample 1 could be explained by the fact that the pores in Sample 1 (208 µm) are bigger than the pores in Sample 3 (172.81 µm). Therefore, the swelling ratio in Sample 3 is lower than the swelling ratio in Sample 1 due to the addition of Ps and the reduced porosity.

The degradation of tissue scaffolds is a critical parameter during the tissue regeneration process [77]. The scaffolds should be able to have an appropriate degradation rate until the new extracellular matrix (ECM) is steady [13]. The degradation tests for Sample 1 and Sample 3 were performed in PBS (pH 7.4) at 37 °C by measuring the weight loss percentage. As shown in Figure 5b, the degradation lasted 7 days (168 h). The weight loss in Sample 1 approached 60% percent and the weight loss in Sample 3 reached nearly 50% percent at the end of 7 days. As in swelling, the highest weight loss percentage was observed in Sample 1. The first reason is the swelling capacity for this situation. Sample 1 had the higher swelling capacity than Sample 3. The samples’ swelling ability causes more freedom for amorphous chains to move easily. If there is no crosslinking, the fibers could be ruptured from the structure by the help of media and accelerate weight loss [13,57]. Therefore, Sample 1‘s chains tend to easily break down during degradation tests due to high swelling capacity even if it was crosslinked. Secondly, the pore structure affects degradation and weight loss rate. There is a direct relationship between the pore size and swelling capacity. Therefore, degradation is observed more rapidly and highly due to the high water absorption ability of scaffolds and weakening of mechanical properties in highly porous structures. Odelius et al. showed that the degradation rate of porous structures decreased with decreasing pore size [78]. Thirdly, the weight loss in Sample 3 resulted from the Ps releasing because hydrolytic degradation accelerates the Ps release with weight loss [74]. Still, this weight loss percentage was not more significant than Sample 1’s weight loss percentage due to highly absorbent properties of SA polymers.

#### 3.2.7. Biological Assessments

**Figure 6:**
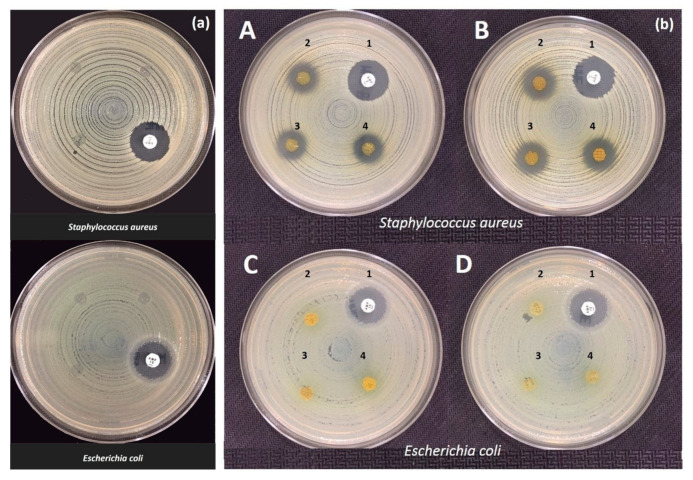
(**a**) The SA without Ps scaffolds against *S. aureus* and *E. coli* after 18 h incubation at 37 °C. (**b**) The inhibition zones of different Ps concentrations and ampicillin (2 and 10 μg) against *S. aureus* and *E. coli* after 18 h incubation at 37 °C. (**A**): Ampicillin 2 μg disk (1), Ps low concentration disks (2–4) with *S.aureus* ATCC^®^ 29213™; (**B**): Ampicillin 2 μg disk (1), Ps high concentration disks (2–4) with *S.aureus* ATCC^®^ 29213™; (**C**): Ampicillin 10 μg disk (1), Ps low concentration disks (2–4) with *E.coli* ATCC^®^ 25922™; (**D**): Ampicillin 10 μg disk (1), Ps high concentration disks (2–4) with *E.coli* ATCC^®^ 25922™.

Antibacterial activity of SA–Ps scaffolds was determined by disk diffusion assay and the results are shown in Figure 6 and Table 5. None of the Ps-incorporated samples could exhibit a zone of inhibition for *E. coli* ATCC^®^ 25922™ strain. Furthermore, SA disks without Ps did not show any antibacterial effect against *E. coli* ATCC^®^ 25922™ and *S. aureus* ATCC^®^ 29213™. Zones measured around ampicillin disks (2 μg and 10 μg) for both bacterial strains were in the expected range according to EUCAST criteria [79]. Moreover, disks containing different concentrations of Ps exhibited significant antibacterial activity against *S. aureus* ATCC^®^ 29213™, which was comparable with ampicillin (Figure 6). These results can be explained taking into account the sensitivity of these strains against Ps and especially to the low solubility of Ps.

The *S. aureus* and *E. coli* bacteria are the most common bacteria types among hospital bacteria. Therefore, nearly all material antibacterial tests were performed with these two types of bacteria. However, not only these two types of bacteria but also *Salmonella spp.* and *P. aeruginosa* are other types used for determining Ps antibacterial activity. The antibacterial activity of Ps against *S. aureus* was proved with some studies. Arıkan et al. studied Ps-added electrospun fabric antibacterial activity against both Gram-positive *(S. aureus)* and Gram-negative bacteria *(A. Baumanni and P. aeruginosa)* [80]. The fabrics displayed antibacterial activity against *S. aureus*, but it did not provide antibacterial activity against Gram-negative bacteria. In another study, Silici et al. showed that ethanolic extract of different Ps samples displayed high antibacterial activity against Gram-positive cocci *(S. aureus)*, however they had a weak antibacterial activity against Gram-negative bacteria *(E. coli and P. Aeruginosa)* [81]. Furthermore, Akca et al. showed that Ps was more effective at inhibiting Gram-positive bacteria than Gram-negative bacteria in their biofilm state [82]. The antibacterial activity of Ps could be considered in two stages [83]. Firstly, it shows a direct effect on microorganisms and secondly, Ps stimulates the immune system, resulting in activation of natural defenses of microorganisms [84]. Ps affects cellular membrane permeability of microorganisms and disrupts membrane potential. It results in reduced adenosine triphosphate (ATP) production and decreasing bacterial mobility. However, the species-specific outer membrane structure of the microorganisms is also another important criteria for presenting the antibacterial activity of Ps. For instance, Gram-negative bacteria have some hydrolytic enzymes that degrade active components of Ps. Therefore, the antibacterial activity of Ps is lower in Gram-negative bacteria than Gram-positive bacteria [85,86]. In this study, the antibacterial activity of Ps was also observed in *S. aureus* strains due to different outer membrane structures of microorganisms. The results were aligned with the literature and therefore Ps is a good candidate and alternative instead of drugs as an antibacterial preservative for wound healing processes.

**Figure 7:**
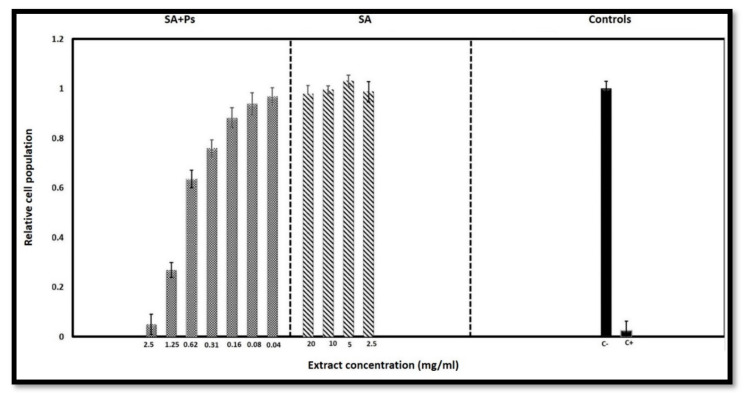
Results of the cytotoxicity test: cell populations calculated relative to the negative control show that extracts obtained with the SA scaffolds are non-cytotoxic for the concentrations tested and extracts obtained with the Ps-containing scaffolds are severely cytotoxic for concentrations of 1.25 mg/mL and above but become non-cytotoxic at concentrations of 0.08 mg/mL and below.

Figure 7 shows the relative cell population for the different extracts and concentrations and the negative and positive cell controls. When relative populations are above 90%, the extracts are considered non-cytotoxic. This happens for all extracts obtained with SA scaffolds, which means that these extracts are non-cytotoxic up to at least a concentration of 20 mg/mL. For the extracts obtained with the Ps-containing scaffolds, the highest non-cytotoxic concentration is 0.08 mg/mL.

Given the Ps to SA mass ratio, this concentration corresponds to Ps concentration of 18.5 µg/mL in the extracts, provided Ps contained in the scaffolds is released to the culture medium. At 0.16 mg/mL the extract is slightly cytotoxic and at 0.31 and 0.62 mg/mL the extracts are moderately cytotoxic. At 1.25 mg/mL (which corresponds to a maximum Ps concentration of 288 µg/mL) and above the extracts are severely cytotoxic. These results are aligned with other results already reported in the literature. Frozza et al. [87] and Silva et al. [88] investigated the in vitro cytotoxic activity of red Ps from Sergipe, Brazil. Frozza et al. found that short term (1 h and 24 h) exposure to hydroalcoholic extracts had inhibitory effects on tumor cells (human laryngeal epidermoid carcinoma, Hep-2) corresponding to IC_50_ values of 128 ± 5 µg/mL and 63.5 ± 3.3 µg/mL, respectively, while for normal cells (human embryonic kidney, Hek-293) the IC_50_ was in excess of 150 µg/mL for both exposure periods [87]. Silva et al. observed that cell exposure to ethanolic extracts over 72 h resulted in IC_50_ values for the glioblastoma (SF-295) tumor cell line of 13.7 ± 2.5 µg/mL and 18.5 ± 3.4 µg/mL for two different Ps lots.

In contrast, colon tumor cells (HCT-116) were less sensitive to Ps extracts, with IC_50_ values ranging from 14.4 to 41.6 μg/mL [88]. Calhelha et al. [89] evaluated the cytotoxicity of phenolic extracts from Portuguese Ps of different origins using human tumor cell lines and porcine liver non-tumor primary cells. Growth inhibition was assessed after exposing cells to Ps extracts for 48 h. Cytotoxic doses for tumor and normal cells were in close proximity and typically in the range of 30 to 50 µg/mL except for the HCT15 (colon carcinoma) cells for which GI_50_ values as low as 10 µg/mL were obtained for some of the Ps extracts studied.

Although the Ps concentrations used in the production of scaffolds in this work may lead to the presence of Ps in the wound fluid at concentrations above the inhibitory concentrations reported in the literature and the cytotoxicity threshold determined in this work, it is important to note that the antimicrobial effect is most important and necessary upon dressing application to a wound and as time passes the wound exudates will wash the excess of Ps away from the wound and conduce an environment where cells can proliferate and proceed into the tissue formation and remodeling phases of wound healing.

## 4. Conclusions

In this study, Ps-added SA scaffolds were successfully developed and produced by a modified extrusion 3D printer. Sodium alginate was chosen as a polymer due to its important parameters for tissue engineering applications. Propolis was used as an active biological substance that has a high potential for wound healing application because of its antimicrobial effects. Different concentrations of SA and Ps were mixed and the optimum concentrations were determined according to physicochemical parameters for the 3D printing process. The optimized scaffold was determined as Sample 3 (4.5 wt % SA and 20 *v/v* %, propolis) and the experiments were presented on this combination. The highest mechanical properties were observed in Sample 3. The desired morphological and biological properties were also obtained in Sample 3. The scaffold pore size of Sample 3 was found to be 172 µm, which means that it is, according to the literature, a proper pore size for skin tissue engineering, i.e., the required pore size of fibroblast cells binding for wound healing. The antimicrobial results were close to well-established ampicillin antibiotics as a control substance. Moreover, cell cytotoxicity analyses were studied. The results showed that the Ps-added scaffolds were non-toxic at low concentrations. Considering the results produced in this study, the 3D-printed Ps-reinforced SA scaffolds have promising properties and they can be employed as biomaterials for further tissue engineering applications.

## Figures and Tables

**Table 1:** The contents of each sample. SA, sodium alginate; Ps, propolis.

Prepared Solutions	SA Content (wt %)	Ps Content (*v*/*v* %)
Sample 1	4.5	0
Sample 2	4.5	10
Sample 3	4.5	20
Sample 4	4.5	40

**Table 2:** Physical properties summary of scaffolds.

Samples	Viscosity (mPa.s)	Density (kg/m^3^)	Surface Tension (mN.m^−1^)	Image of 20 Layers Printed Scaffold
Sample 1	6109	1.022 ± 0.02	39.43	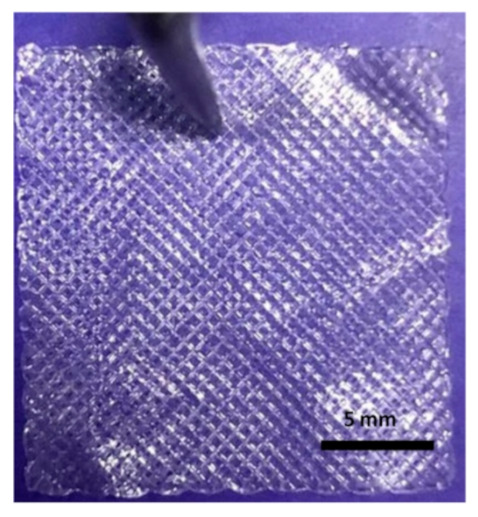
Sample 2	8018	1.023 ± 0.03	44.73	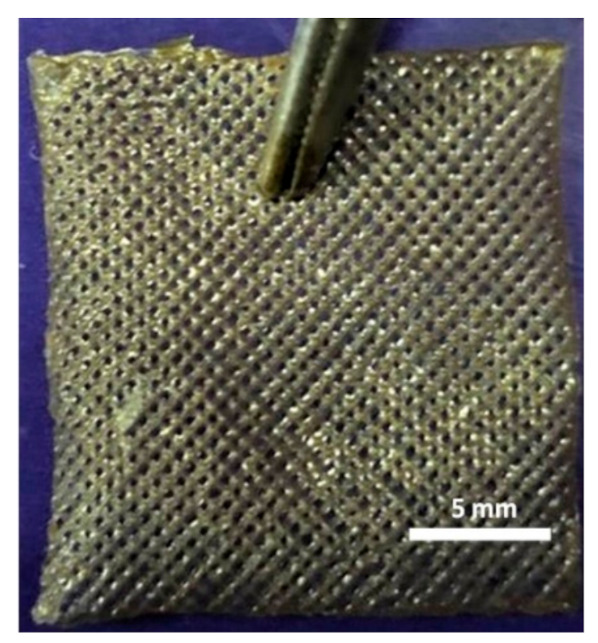
Sample 3	8028	1.024 ± 0.02	64.78	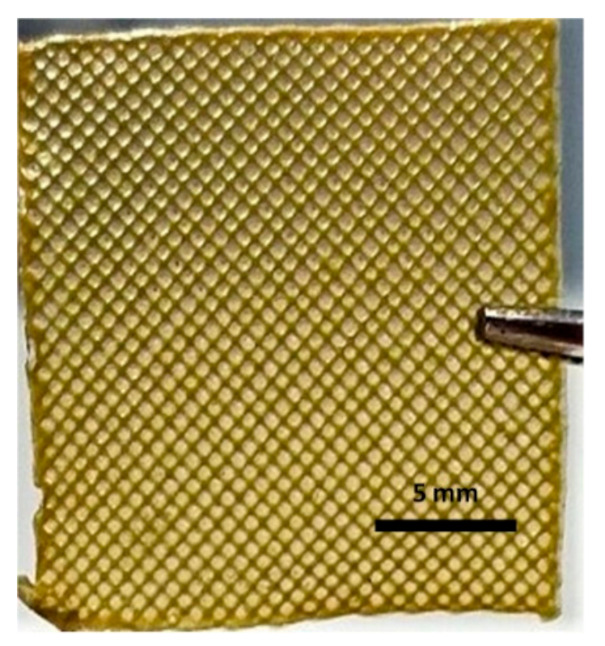
Sample 4	Measurement failed	Measurement failed	Measurement failed	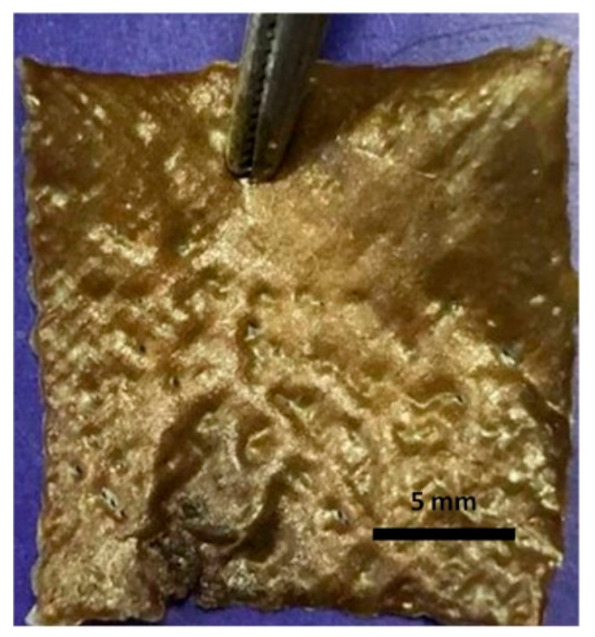

**Table 3:** Tensile test measurements of produced scaffolds.

Scaffolds	Tensile Strength (MPa)	Elongation at Break (%)
Sample 1	3.92 ± 1.08	14.74 ± 1.22
Sample 2	2.42 ± 0.62	20.57 ± 1.96
Sample 3	2.33 ± 1.76	25.00 ± 6.68

**Table 4:** Fitting experimental release data from the Ps release of 3D-printed scaffolds to Higuchi and Korsmeyer–Peppas kinetic equations for neutral and acidic conditions (pH 7.4 and 2).

Release pH	Higuchi Model		Korsmeyer–Peppas Model	
	R^2^	K_h_	R^2^	n
pH 7.4	0.9822	6.025	0.9715	0.4492
pH 2.0	0.9515	7.9228	0.8633	0.6153

**Table 5:** Inhibition zone measurements of *S. aureus* and *E. coli* obtained with different Ps concentrations and ampicillin (2 and 10 μg).

	Inhibition Zone (mm)				
Bacteria	SA ^†^	Ps ^‡^ Low	Ps High	Ampicillin 2	Ampicillin 10
*S. aureus*	0	15.00 ± 1.1	13.00 ± 1.0	18.00 ± 1.0	NA
*E. coli*	0	0	0	NA	19.00 ± 1.1

† SA, sodium alginate; ‡ Ps, propolis.

## Abbreviations

3D	Three dimensional
AM	Additive Manufacturing
DMEM	Dulbecco’s Modified Eagle’s Medium
DMSO	Dimethyl sulfoxide
FBS	Fetal Bovine Serum
FT-IR	Fourier Transform-Infrared Spectroscopy
HFFF2	Human Dermal Fibroblasts
PBS	Phosphate-Buffered Saline
Ps	Propolis
SA	Sodium Alginate
SBS	Scientific Bio Solutions
SD	Standard deviation
SEM	Scanning electron microscopy
STL	Stereolithography
TGF-β	Transforming Growth Factor-β
